# Mechanical properties and microstructural change of W–Y_2_O_3_ alloy under helium irradiation

**DOI:** 10.1038/srep12755

**Published:** 2015-07-31

**Authors:** Xiaoyue Tan, Laima Luo, Hongyu Chen, Xiaoyong Zhu, Xiang Zan, Guangnan Luo, Junling Chen, Ping Li, Jigui Cheng, Dongping Liu, Yucheng Wu

**Affiliations:** 1 School of Materials Science and Engineering, Hefei University of Technology, Hefei 230009, People’s Republic of China; 2 Institute of Plasma Physics, Chinese Academy of Sciences, Hefei 230031, People’s Republic of China; 3 National–Local Joint Engineering Research Centre of Nonferrous Metals and Processing Technology, Hefei 230009, People’s Republic of China; 4 School of Physics and Materials Engineering, Dalian Nationalities University, Dalian 116600, People’s Republic of China

## Abstract

A wet-chemical method combined with spark plasma sintering was used to prepare a W–Y_2_O_3_ alloy. High-temperature tensile tests and nano-indentation microhardness tests were used to characterize the mechanical properties of the alloy. After He-ion irradiation, fuzz and He bubbles were observed on the irradiated surface. The irradiation embrittlement was reflected by the crack indentations formed during the microhardness tests. A phase transformation from α-W to γ-W was investigated by X-ray diffraction (XRD) and transmission electron microscopy (TEM). Polycrystallization and amorphization were also observed in the irradiation damage layer. The W materials tended to exhibit lattice distortion, amorphization, polycrystallization and phase transformation under He-ion irradiation. The transformation mechanism predicted by the atomic lattice model was consistent with the available experimental observations. These findings clarify the mechanism of the structural transition of W under ion irradiation and provide a clue for identifying materials with greater irradiation resistance.

W and its alloys are used as plasma facing materials (PFMs) in the International Thermonuclear Experimental Reactor (ITER) and exhibit promise as materials for use in fusion devices because of their excellent properties, such as their high melting points, good high-temperature strength, high sputtering thresholds and high irradiation resistance^1^,^2^,^3^. However, these alloys exhibit serious embrittlement problems at low temperatures during recrystallization and irradiation^4^,^5^,^6^,^7^. On the one hand, low-temperature embrittlement is associated with a high ductile-to-brittle transition temperature (DBTT). Some authors^8^,^9^,^10^,^11^ have demonstrated that fine oxide particles dispersed in the W matrix can improve the embrittlement values through a decrease of the DBTT and an increase of the recrystallization temperature (RCT). In fusion reactors, plasma-facing materials exhibit an operating temperature window in the range from 800 to 1200 °C as the DBTT and RCT limits^11^,^12^. At operating temperatures below 800 °C, the DBTT of the material is required under fusion specific conditions.

On the other hand, irradiation embrittlement caused by the implantation of these energetic particles (H, D, T, He and neutrons) into the first wall results in irradiation defects upon fusion reactor operation^13^,^14^,^15^,^16^. In general, PFMs used in a fusion reactor suffer from two types of damage: displacement damage due to high-energy neutrons and surface damage due to hydrogen and helium from the plasma^17^. To understand the effect of these energetic particles on surface modification of W, researchers have performed various irradiation experiments (e.g., Fe, He, D, H ion, neutron)^18^,^19^,^20^,^21^,^22^,^23^.

A number of studies have been focused on the irradiation damage of W-matrix materials. The literature also contains numerous reports on irradiation-induced morphological damage and the underlying mechanisms at the macro level. The mechanism for H-blister formation in W was investigated by Mateus *et al.* and by Lu *et al.*^24^,^25^. The morphology of nanostructure fuzz has been widely and deeply studied^26^,^27^,^28^,^29^,^30^,^31^. Kajita *et al.* proposed a schematic of ion-irradiation damage to illustrate the process of the formation of fuzz^30^. The formation mechanism of the He clustering and bubble were studied in depth, which revealed that the formation mechanism is induced by one point defect (interstitial atoms, vacancies) or grain boundary trapping He atoms under He-ion irradiation^31^,^32^. On the other hand, other works have been focused on the evolution of the irradiation-induced atomic-scale changes of the lattice and crystal structure. The irradiation-reduced microstructure transformation at the atomic level was also studied using transmission electron microscopy (TEM)^33^,^34^,^35^,^36^. Wang *et al.*^33^ observed that the grain boundaries of W/ZrO_2_ are perturbed, which may be a consequence of point defects induced by changes of the atomic configuration. The phase transformation has also been studied by various groups^34^,^35^,^36^. To study the behavior of these irradiation defects (the interstitial atoms, vacancy), other researchers have studied the formation energy and the binding energy using simulation calculations^37^,^38^.

Studying the He-irradiation behavior of W is important because He is an important fusion product produced in D and T fusion reactors. Irradiation damage acts as a simulation of particle bombardment. Moreover, the migration and accumulation of deposited He ions can lead to the formation of He bubbles in materials, which can induce serious volume swelling^39^ and embrittlement^13^,^14^,^15^,^16^. Therefore, structural damage induced by He is a significant issue for nuclear materials. In the present work, W–Y_2_O_3_ alloy is studied via high-temperature tensile tests and nano-indentation tests. The microstructure and microhardness of the materials before and after irradiation are studied in detail. Two models based on experimental work are proposed to explain the process of the phase transformation.

## Results

### Sample characterization

**Figure 1:**
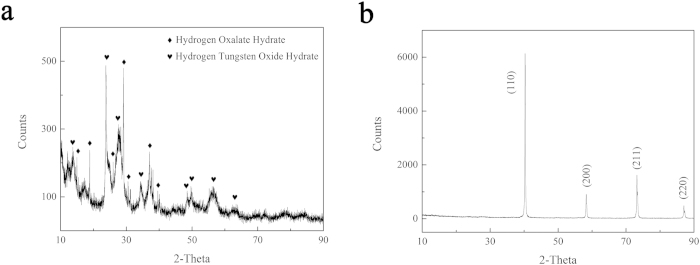
(**a**) XRD pattern of the precursor of W–Y_2_O_3_; (**b**) XRD pattern of the W–Y_2_O_3_ powder after reduction.

Figure 1 shows the X-ray diffraction (XRD) pattern of the W–Y_2_O_3_ powder before and after reduction. As shown in Fig. 1a, the obtained precursor consists of hydrogen oxalate hydrate (JCPDS#14-0832) and hydrogen tungsten oxide hydrate (JCPDS#40-0693), which are attributable to the residual hydrogen oxalate hydrate and the precursor of tungsten. Figure 1b shows that the reduced powder is pure W. The lattice constant of *a* = 3.1644 Å calculated from the diffraction data is consistent with data reported in JCPDS #04-0806 (*a* = 3.1648 Å). However, no Y_2_O_3_ peak was detected in the XRD pattern, which may be attributable to the low ratio of added Y_2_O_3_.

**Figure 2:**
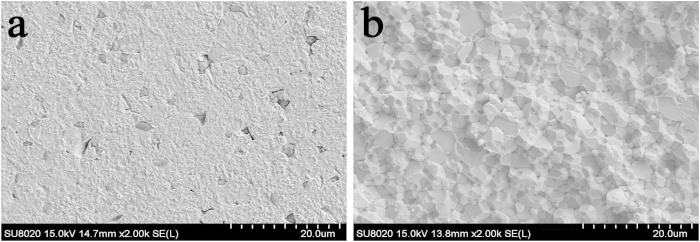
SEM images of the W–Y_2_O_3_ alloy: (**a**) polished and etched surface structures; (**b**) fracture morphology at RT.

The sample exhibited a high density after SPS sintering. The actual density was 18.79 g/cm^3^, which is very close to the calculated theoretical density (18.97 g/cm^3^), demonstrating a relative density of 99.05%. An SEM image of a polished surface is displayed in Fig. 2a. Y_2_O_3_ was observed to be distributed at the grain boundaries (2000 × magnification). As shown in Fig. 2b, the fracture surface of the W–Y_2_O_3_ exhibited room-temperature intergranular fracture and some transgranular fractures. As shown in the figure, the mechanism of failure was brittle fracture.

### Mechanical properties

**Figure 3:**
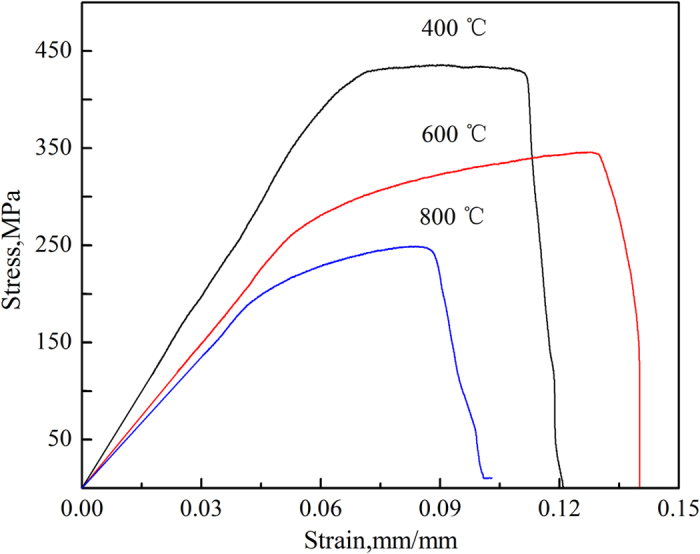
Stress–strain curves of the W–Y_2_O_3_ alloy measured at different temperatures.

To investigate the mechanical properties of the W–Y_2_O_3_ alloy, we subjected it to tensile and nano-indentation tests. Figure 3 shows the stress as a function of strain at temperatures of 400, 600 and 800 °C. The tensile strength of the W–Y_2_O_3_ alloy decreased with increasing temperature. The yield strength, tensile strength, elongation and elastic modulus determined from Fig. 3 are presented in Table 1. The yield strengths (*δ*_0.2_) at temperatures of 400, 600 and 800 °C were 397.4, 273.1, and 206.4 MPa, respectively. Accordingly, the elongations of the alloy at temperatures of 400, 600 and 800 °C were 6.4%, 7.1%, and 3.9%, respectively. The highest tensile strength of 436 MPa for the W–Y_2_O_3_ alloy was obtained at 400 °C, and the highest elongation of 7.1% was obtained at 600 °C. In general, elongation should increase with increasing temperature. However, an abnormal elongation occurred at 800 °C; this elongation could be reduced by high-temperature tension under a non-protective atmosphere owing to the oxidation of the alloy. The curve indicates that the W–Y_2_O_3_ alloy exhibited evident plasticity and implies that the DBTT of this alloy is below 400 °C. The nano-indentation tests were performed at six points. The average Berkovich hardness and average Young’s modulus of the W–Y_2_O_3_ alloy were 6.56 and 371 GPa, respectively. The hardness value of W–Y_2_O_3_ is greater than those of pure W and W–2 wt.%Y_2_O_3_ reported in the literature^8^; however, the Young’s modulus of the W–Y_2_O_3_ alloy is lower than that of W–2 wt.% Y_2_O_3_ and greater than that of pure W.

### Characterization of irradiated W–Y_2_O_3_ alloy

**Figure 4:**
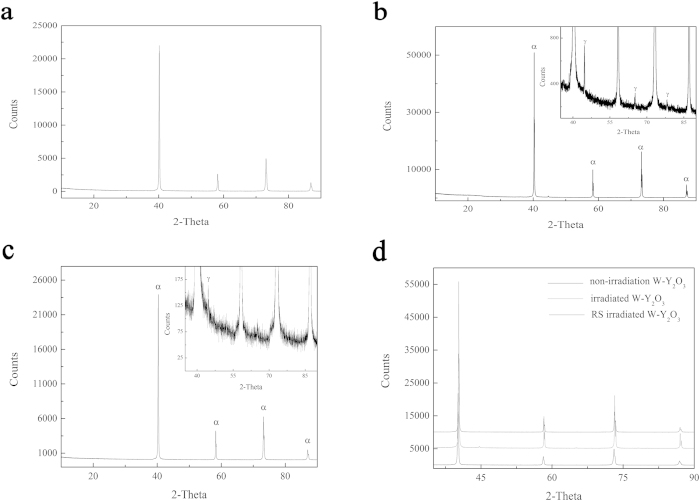
XRD patterns of the W–Y_2_O_3_ alloy before and after irradiation: (**a**) non-irradiated W–Y_2_O_3_ sample; (**b**) irradiated W–Y_2_O_3_ sample; (**c**) removed surface of the irradiated W–Y_2_O_3_ sample; (**d**) the three above-mentioned XRD patterns illustrated in the same image.

XRD was used to characterize the non-irradiated, irradiated, and removed surface (RS) of the irradiated W–Y_2_O_3_ (RS-irradiated W–Y_2_O_3_). Figure 4a shows that the non-irradiated W–Y_2_O_3_ alloy with space group Im–3 m (229) has a lattice parameter of 3.1712 Å, which well matches that of the body-centered cubic (bcc) α-W phase (JCPDS #04-0806). Figure 4b shows that the irradiated W–Y_2_O_3_ alloy contains α-W and γ-W phases (the XRD pattern of γ-W is shown in the inset of Fig. 4b, and its space group is Fm–3 m (225) (JCPDS #88-2339)). When the back irradiated surface was removed (a back layer on the surface could be viewed by the naked eye after irradiation.), the γ-W peaks decreased in intensity and number, with only 164 counts (Fig. 4c inset), in comparison with those of irradiated W–Y_2_O_3_, with a corresponding γ-W peak intensity of 780 counts (Fig. 4b inset). These results imply that the W–Y_2_O_3_ alloy undergoes a phase transformation from the α-W phase to the γ-W phase under He-ion irradiation and that the phase transformation mainly occur at the back-surface irradiation damage area. In general, W possesses a stable bcc structure with space group of Im–3 m (229) and a lattice parameter of *a* = 3.1648 Å. Sufficient activation energy is required to overcome the energy barrier of phase transformation. Given that ion irradiation can introduce a large number of defects, the phase transformation observed in ion-irradiated W is most likely induced by irradiation-induced high stress. Similar ion-irradiation-induced phase transformations have been observed in other materials, including Ti_3_AlC_2_, V and Y_2_O_3_^34^,^35^,^36^. The structural transformation is accompanied by polygonization and the formation of nanograins. The transformation process of the irradiated W phase from a bcc structure to an fcc structure will be discussed in a later section on the basis of high-resolution transmission electron microscopy (HRTEM) analysis. In addition, the XRD peak intensity of irradiated W–Y_2_O_3_ is considerably lower than that of non-irradiated and RS-irradiated W–Y_2_O_3_. As shown in Fig. 4d, the peaks of the irradiated and RS-irradiated W–Y_2_O_3_ did not substantially deviate from each other; however, they both exhibited a slight right shift of *2θ* = 0.15° compared to the peak of the non-irradiated sample, probably as a result of irradiation-induced stress.

**Figure 5:**
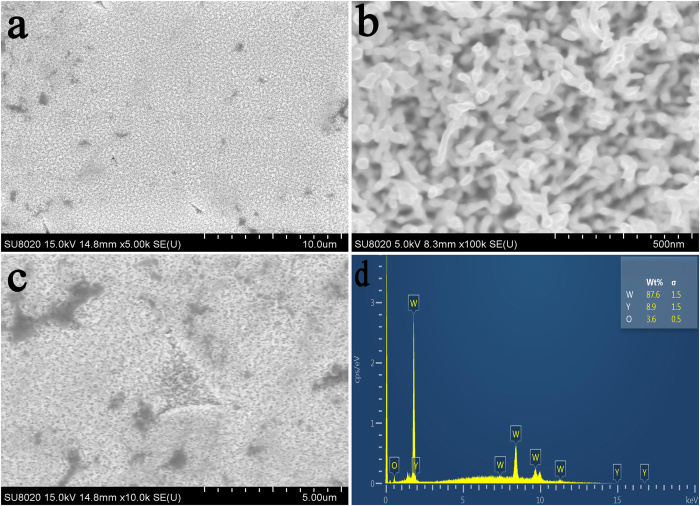
(**a**) SEM image of the irradiated W–Y_2_O_3_ sample at low resolution; (**b**) SEM image of the fuzz structures at high resolution; (**c**) SEM image of a pit located at the trigeminal grain boundary; (**d**) corresponding EDS spectrum of the pit shown in (**c**).

Figure 5 shows the top view of the W–Y_2_O_3_ alloy after irradiation. A high-magnification image is presented in Fig. 5b, which shows a fuzzy morphology. The nanostructural fuzz is the same fuzz reported elsewhere^26^,^27^,^28^,^29^,^30^. In general, Y_2_O_3_ particles should be observed at a magnification of 5000×, as shown in Fig. 5a. However, only several Y_2_O_3_ pits clearly located at the trigeminal grain boundary are observed in Fig. 5a,c. The pits are clearly located at the trigeminal grain boundary, which is known to be the site of Y_2_O_3_ particles, as shown in Fig. 2a. In addition, the energy-dispersive X-ray spectroscopy (EDS) spectrum in Fig. 5d further confirms the presence of Y_2_O_3_.

**Figure 6:**
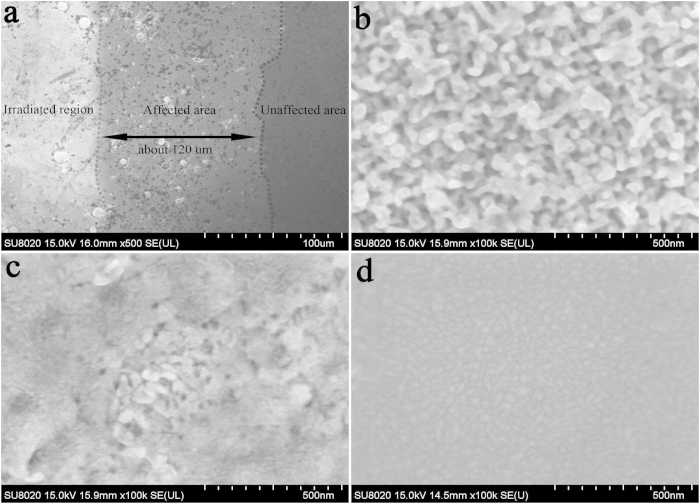
(**a**) The images of the interface consisting of the irradiated region, the affected area, and the unaffected area; (**b**) high-resolution image of the irradiated region; (**c**) high-resolution image of the affected area near the irradiated region; (**d**) high-resolution image of the affected area near the unaffected area.

The dimensions of the samples for He-ion irradiation experiments were 10 × 10 mm^2^, and the effective He-ion beam was incident onto a circular region 10 mm in diameter. Thus, an obvious interface was present at the surface of the sample after He-ion irradiation. Figure 6a focuses on the interface, which separates the irradiated areas from the non-irradiated areas. The red dotted line is the boundary line between the irradiated region (left) and the affected area (right). The irradiated region was the area in which He ions impinged directly; the surface temperature at this region was 1377 ± 10 K as a result of the He-ion irradiation. The affected area was the area that exhibited no exposure to He-ion irradiation, but whose morphology was thermally affected. The blue dotted line is the boundary line, which separates the unaffected area from the affected area. From Fig. 6a, the affected area was approximately 120 μm. Figure 6b displays the surface of the irradiated region with a fuzzy nanostructure. Figure 6c shows a small and dense nanostructure at the heavily heat-affected area. Several nanosized bulges are observed in Fig. 6d at the weakly heat-affected area. High-power laser irradiation of W materials also has also been reported to result in a nanostructured morphology on their surface^40^,^41^. This previous work, the morphology of the nanostructures was related to the temperature increase, with the nanostructures being formed on the surface mainly as a result of non-uniform surface heating^42^. The affected area was also subjected to the temperature and field of some nanostructures on the surface. Given that the temperature was maximal at the irradiated region and decreased toward the unaffected area, a temperature gradient existed, resulting in different morphologies along the irradiated region to the unaffected area. Thus, this difference in surface morphology at the affected area is attributable to the decreased thermal effects from the irradiated region to the unaffected area.

**Figure 7:**
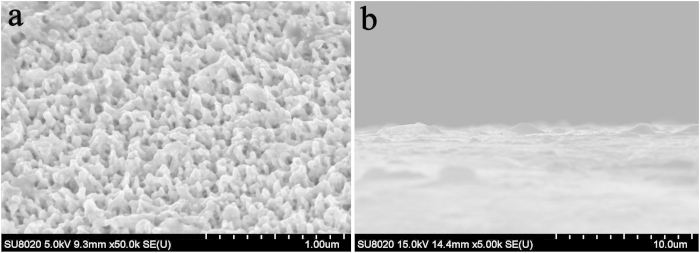
(**a**) Image of the fuzz structures viewed from the tilted sample; (**b**) image of a layered structure viewed from the cross-section of the sample.

The depth of irradiation effects was also studied. The irradiated sample was placed on a tilting sample stage and characterized by FESEM, as shown in Fig. 7a. The fuzz size and length were observed to be approximately 50 and 100–200 nm, respectively. The sample was broken with pliers, which enabled observation of the cross-section of the irradiation region. Figure 7b shows the cross-sectional view, which displays a layered structure with a thickness of approximately 2 μm. In addition, several bubbles are evident in the layered structure. The structure of the damaged region consists of the surface with nanosized fuzz and a subsurface with a layered, thin, damaged structure. These damaged W–Y_2_O_3_ alloy surfaces may be the reason for the lower peak intensity of the irradiated sample (Fig. 4d).

The fuzz formation sheds light on the improvement of irradiation resistance. The incident ion energy and the surface temperature are virtual conditions for the formation of the nanostructures^28^−^30^. The fuzz has been identified as nanometer-sized He gas bubbles in a tendril^30^,^31^. Kajita *et al.*^30^ provided schematics of the nanostructure formation process and, on the basis of simulation results^43^, proposed that the size of the initially formed holes increases with increasing surface temperature. Because the temperature of our specimen surface was 1377 K, the initial hole structures were formed. Subsequently, the holes grew along the depth direction by coalescence of the hole and the bubbles, resulting in the formation of the nanostructured fuzz. However, Lasa^44^ and Sefta *et al.*^45^ proposed that the formation of these pinholes was due to the growth and rupture of He bubbles. He bubbles form more easily in these holes and will also rupture more easily. The fuzz structure would be formed under further He irradiation.

Microhardness was used to investigate the W–Y_2_O_3_ alloy. As shown in Table 2, the microhardness decreased after He-ion irradiation. However, the values only slightly diverged in the case of the removal of the damaged surface, which implies that the alloy surface and the subsurface were affected by He-ion irradiation. In the case of the irradiated sample, the surface was loose and exhibited a fuzz structure. In addition, certain bulk defect-like cavities, clustering and bubbles could lead to the production of crevices between the subsurface and the substrate, finally resulting in a decrease of the microhardness. When the damaged layer is removed, the surface exhibits its original morphology, which less strongly influences the microhardness. The average value of the microhardness was observed to decrease from 365.8 to 328.1 MPa after irradiation, which is attributable to the thermal stress reduction by the temperature gradient between the irradiated region and the unaffected area.

**Figure 8:**
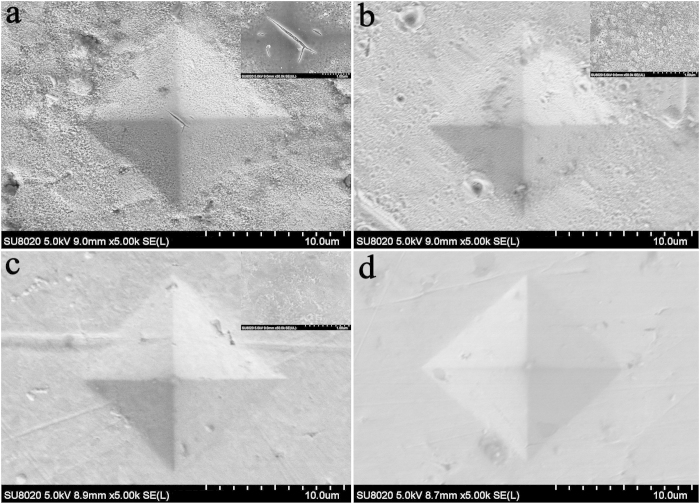
SEM images of the indentations at different regions (the insets are the corresponding high-resolution SEM images). (**a**) irradiated region; (**b**) affected area near the irradiated region; (**c**) affected area near the unaffected area; (**d**) unaffected area.

The indentations resulting from the microhardness tests in the irradiated region, the affected area and the unaffected area were characterized by FE-SEM. Figure 8a shows the indentation of the irradiated region with a loose surface and a crack at the center (inset image). Figure 8b,c show the affected region near the irradiated region and the unaffected area, respectively. The inset displays high magnification images of selected in the vicinity of the indentations. Evidently, these structures near the irradiated region are smaller and denser than those near the unaffected area, as shown in Fig. 6c,d. Figure 8d shows a normal indentation in the unaffected area. In general, irradiation embrittlement or hardening occurs in damaged surfaces^13^−^16^. The irradiation-induced defects act as obstacles that hinder the motion of the dislocations. Thus, the irradiated surface layer and the matrix exhibit different deformation behaviors under the microhardness tests, resulting in an evident crack in the indentation at the irradiation region, as shown in the inset image of Fig. 8a.

**Figure 9:**
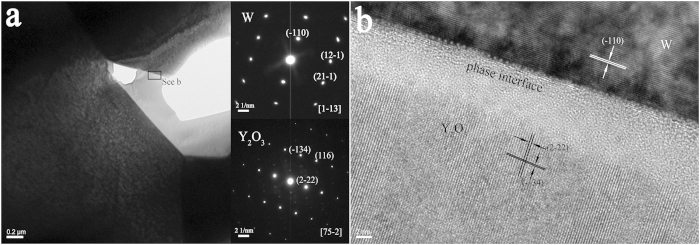
(**a**) A bright-field image of the non-irradiated W–Y_2_O_3_ sample; (**b**) HRTEM image of the phase interface selected from image (**a**).

To elucidate the microstructure, we used TEM to characterize the W–Y_2_O_3_ alloy before and after irradiation. Figure 9a shows a bright-field image of the non-irradiated sample, which shows the Y_2_O_3_ particles distributed at the grain boundary. The corresponding selected-area electron diffraction (SAED) patterns (inset in Fig. 9a) were used to further verify different phases. The W phase is a bcc structure that exists along the zone axis of [11−3]. The SAED patterns along the [75−2] zone axis from the Y_2_O_3_ grain were indexed as a cubic structure. Figure 9b shows the HRTEM image of the phase interface between W and Y_2_O_3_; this image was collected at the area labeled “see b” in Fig. 9a.

**Figure 10:**
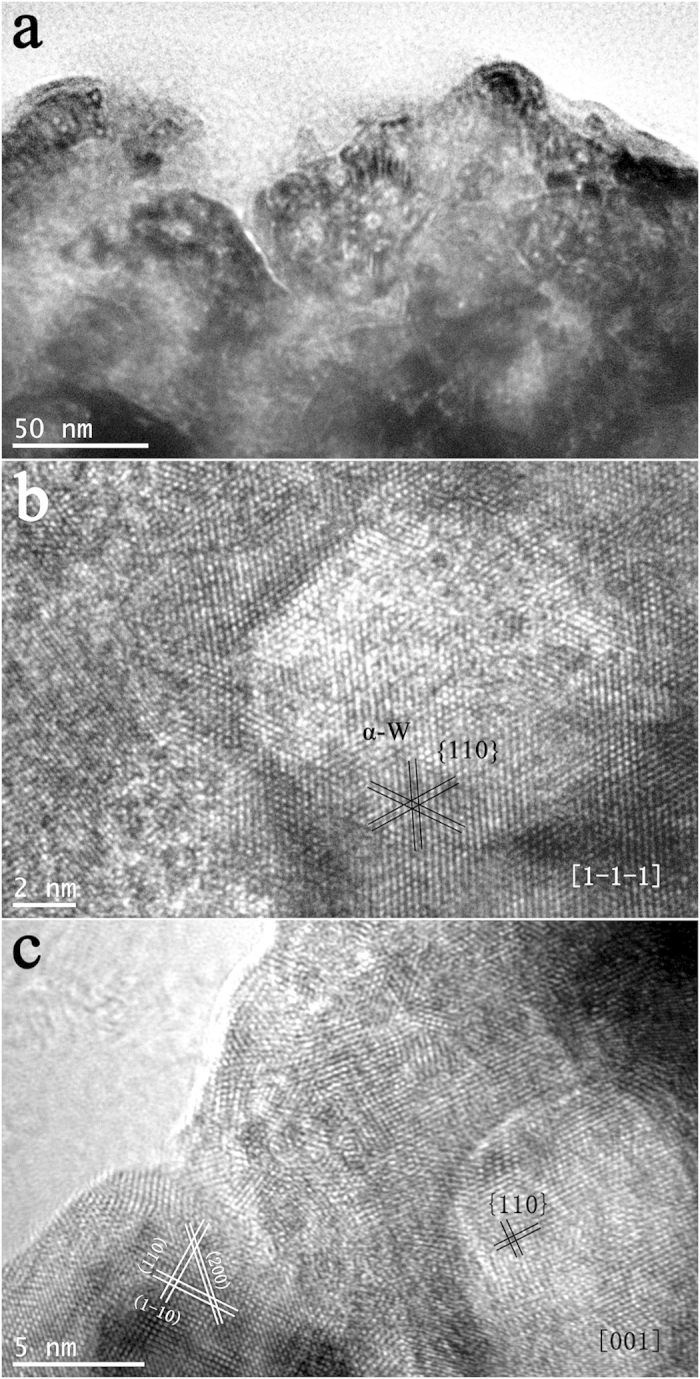
(**a**) A typical bright-field TEM image of the irradiated W–Y_2_O_3_ sample; (**b**) and (**c**) HRTEM images of the two He bubbles detected from (**a**).

The material structure undergoes dramatic changes under He-ion irradiation. We observed the microstructure of the irradiated sample by TEM to determine the influence of the pristine W structure on the kinetics of ion-irradiation-induced structure transformations. Figure 10a shows a typical bright-field image of the irradiation damage surface, which exhibits several hole structures. These hole structures are consistent with the HRTEM results shown in Fig. 10b. The bubble size is approximately 10 nm (this hole structure is referred to as the “He bubble”)^29^. A portion of the crystal lattice has been distorted specifically, the bcc structure of α-W, which was identified from the non-distorted lattice. The crystal planes were {110}, and the HRTEM image was collected along the [1−1−1] zone axis. The results thus imply that a He bubble existed at the crystal plane of (1−1−1). Figure 10c presents another He bubble, showing that He bubbles existed at the (001) crystal plane and that the orientation changed relative to the original orientation of the sample.

A larger number of defects should be produced under higher-fluence He-ion irradiation conditions. He atoms at different sites in W exhibit different solution energies. The configurations for a He atom in W are a vacancy/substitution site (SS), an octahedral interstitial site (OIS) and a tetrahedral interstitial site (TIS). A vacancy can provide a larger space for He; thus, a He atom is more inclined to aggregate at a vacancy than at an interstitial site^46^. Moreover, He atoms are energetically favorable for setting a vacancy/SS, and a TIS is more favorable than an OIS^46^,^47^. At high temperature (1377 K), these defects have sufficient energy to migrate, which could result in the formation and growth of He bubbles^43^,^45^. He atoms are trapped at the vacancies and form stable vacancy–He complexes. Next, these complexes tend to form bubbles at high temperatures. Strong interactions occur among He atoms, resulting in the formation of He clusters^48^,^49^,^50^. In addition, the small clusters act as nucleation sites for the formation of larger He bubbles^31^,^46^. He clusters or He bubbles would relieve the high internal pressure^46^,^47^, which could result in lattice distortion surrounding the He bubbles, as shown in Fig. 10b,c. In the case of the bcc structure of W, the {110} planes are of prime importance because these {110} planes are close-packed with low surface energy. For the interstitial position, He atoms easily agglomerate in a close-packed arrangement between the (110) planes, forming a He monolayer structure; subsequently, He nucleates at the (110) plane and forms a bubble^46^. This mechanism implies that the bubble exists at the (110) planes. However, He bubbles exist at both the (111) and (100) planes, as shown in Fig. 10b,c. These phenomena can be explained by two rational interpretations. First, the same tetrahedral interstitial sites (six positions in a unit cell) exist at the interplane of (110) and (111). The detachment at (111) planes would be possible when He atoms occupy these positions and then form a He bubble. Second, different crystal planes have been confirmed in HRTEM images along the different zone axes. Given that the TEM cannot exhibit morphological contrast, even a He bubble at the (110) crystal plane is not observed, nor is the zone axis of [110].

**Figure 11:**
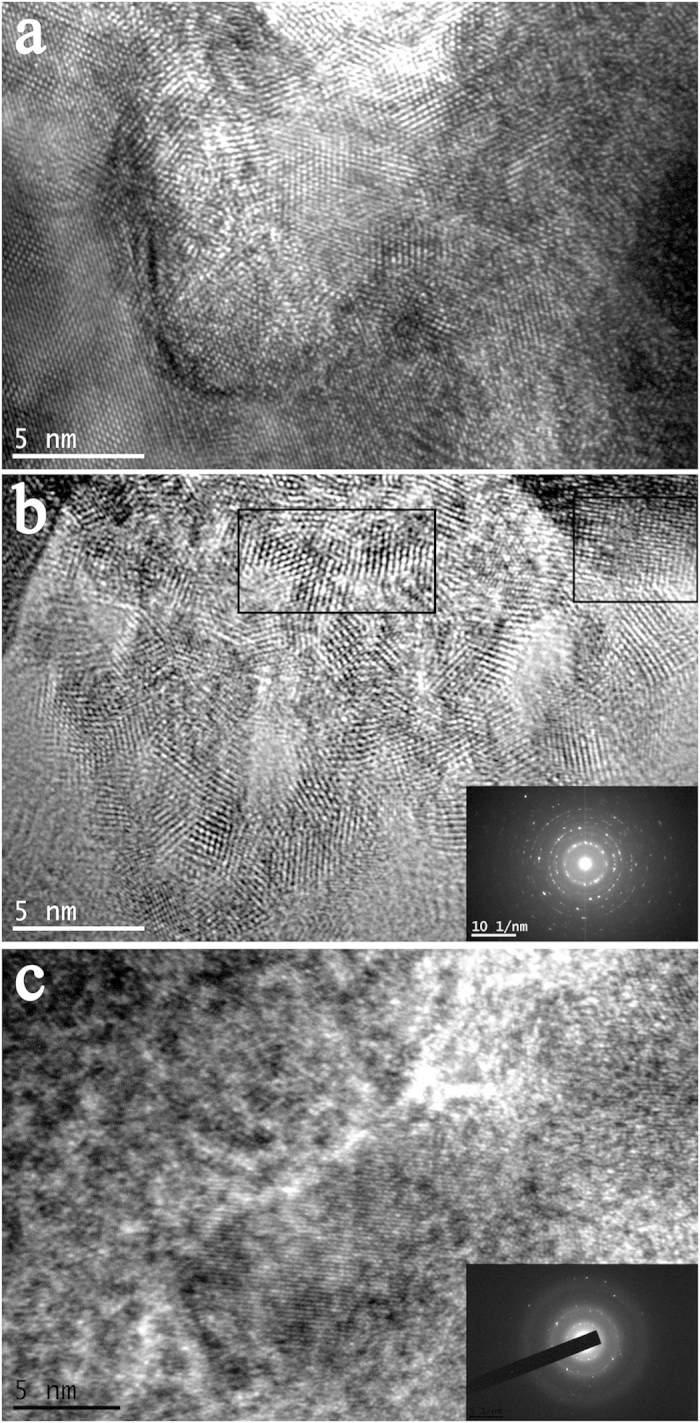
Typical HRTEM images. (**a**) different degrees of distortion of the crystal lattice produced by non-uniform irradiation intensity; (**b**) HRTEM image of the polycrystalline structure; (**c**) HRTEM image of the amorphous structure.

Figure 11 shows some typical HRTEM images of the irradiation-damaged area. In Fig. 11a, part of the crystal lattice retains its original structure and most lattices have been distorted under He-ion irradiation. Different irradiation intensities could lead to different degrees of lattice distortion. As shown in Fig. 11b, a polycrystalline structure with a scattering ring indicates that polycrystallization occurred in the irradiated samples. Figure 11c shows an amorphous structure, and the SAED patterns of the structures in the HRTEM images are presented in the insets. A halo or ring is observed in the SAED patterns, indicating that amorphization occurred in the irradiated samples. From the empirical potential, different degrees of distortion are caused by non-uniform intensity, and non-uniform irradiation intensity is reflected in the He particles that bombard the material on considerably smaller scales. In general, with increasing irradiation intensity and increasing structural change from a normal lattice to a distorted lattice, polycrystalline and amorphous structures are observed. The amorphous structures could produce relatively high-intensity irradiation positions.

## Discussion

### Phase transformation

**Figure 12:**
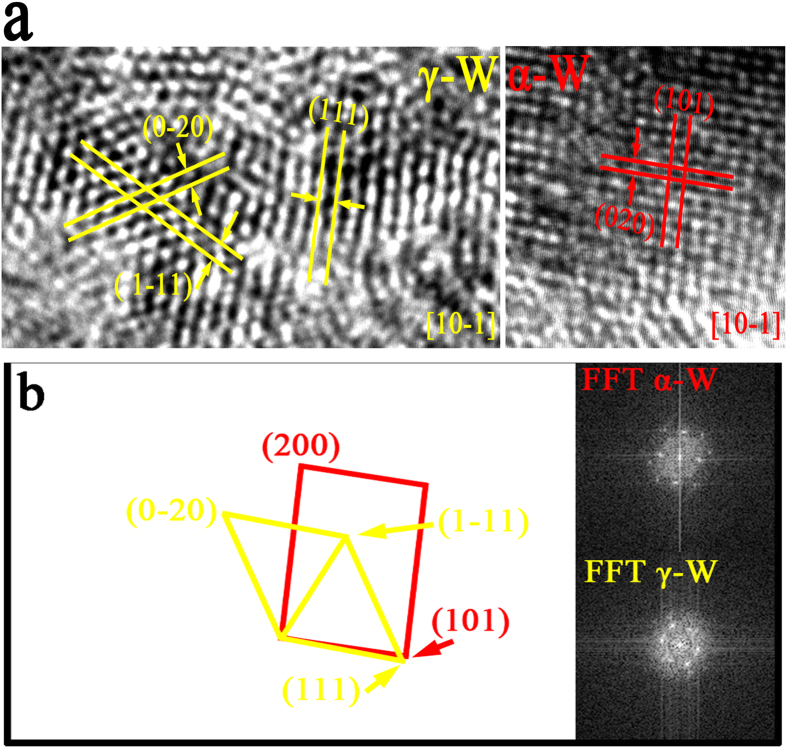
(**a**) HRTEM image of α-W and γ-W selected from Fig. 11b illustrating one process of phase transformation; (**b**) the habitus relationship between α-W and γ-W from the FFT patterns.

He-ion irradiation could promote lattice-structure distortions and a tendency to form polygonal and amorphous structures. In-depth study of the polycrystalline structure showed that this lattice structure was mainly distorted and changed in the grain orientation during the observed phase transformation. As shown in Fig. 12a, two lattice structures were selected from the black rectangular area in Fig. 11b. The region of the left black rectangle is γ-W, as determined from the interplanar spacing and the angles of planes. The area of the right black rectangle corresponds to α-W, as also determined from the interplanar spacing and plane angles. As evident from the results in Fig. 12a, the α-W transformed into the fcc structure of γ-W under He-ion irradiation conditions, and the crystal lattice exhibited a certain degree of distortion due to the increase in interplanar spacing. In Fig. 12b, the insets show the FFT patterns from the selected black rectangular regions. The red and yellow lines denote the amplified FFT patterns of α-W and γ-W, respectively. The average misorientation angle is approximately 5.1° with respect to the main crystallographic direction, which is derived from the distortion of the FFT patterns; i.e., the (111) plane γ-W of is almost parallel with the (101) plane α-W of, indicating that the γ-W phase nucleated on the (101) planes of the α-W phase. Notably, the HRTEM images of the α-W and γ-W phases were collected at the same incident electron beam intensity and along the same [10−1] zone axis. An obvious crystallographic relationship exists between α-W and γ-W, which follows the relation {101}_α_//{111}_γ_. The process follows the empirical potential, which will be clarified later.

**Figure 13:**
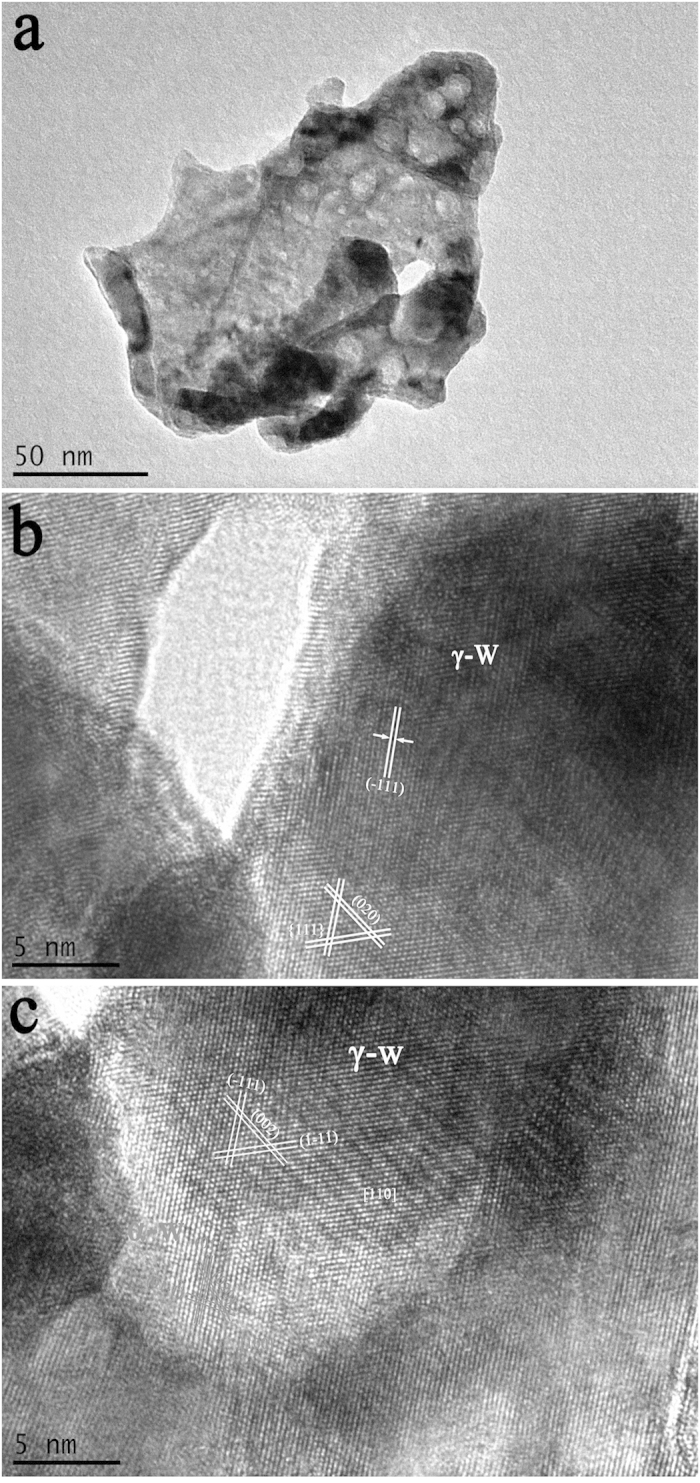
(**a**) A bright-field TEM image; (**b**) and (**c**) HRTEM images chosen from image (**a**) to illustrate another process of phase transformation.

Another phase-transformation process is illustrated in Fig. 13a–c. A narrow void is observed in the bright-field image in Fig. 13a. On the basis of the empirical potential, we propose that the void should be a He bubble that is viewed from the cross-section direction. The surrounding structure of the He bubble was characterized by HRTEM (Fig. 13b). The structure was found to have been changed relative to the stable phase of α-W, becoming the fcc structure of γ-W. Notably, the narrow voids are almost parallel with the plane of (−111), meaning that the He bubbles exist at the (−111) plane corresponding to the γ-W. In Fig. 13c, α-W and γ-W are both observed, and the close-packed plane (−111) of γ-W is parallel to the close-packed plane (011) of α-W. This finding indicates that the α-W phase has transformed to the γ-W phase and that the narrow voids are almost parallel with the plane (011) of α-W, meaning that the He bubbles exist at the (011) plane corresponding to the α-W. The phases of α-W and γ-W are in the same field of view of the HRTEM image with the same incident electron-beam energy and with different zone axes: α-W along [100] and γ-W along [110]. In γ-W, no obvious lattice distortion was observed; however, α-W was transformed into γ-W. We propose that a high internal stress forms in the He bubble and generates a shear strain on the lattice, which induces the phase transformation from α-W to γ-W. With increasing distance from the He bubble, the stress gradually decreases and finally disappears, resulting in the lattice retaining the original bcc-structured α-W phase at the locations away from the He bubble. In Fig. 13c, no obvious interface is observed between α-W and γ-W, which further supports are previous hypothesis. The detailed phase-transformation process is clarified in the next section.

### Process of phase transformation

Two questions have been raised on the basis of the above-mentioned results. Why does the irradiated W transform and grow into an fcc-structured phase? What is the atomic mechanism involved in the He-ion-irradiation-induced phase transformation?

When He ions irradiate the W matrix, stress must form as a result of irradiation-induced defects; this stress increases with increasing radiation dose. Lattice distortion, polycrystallization and amorphization are different stress release methods. Phase transformation also serves as an effective method to release stress because of the migration of W atoms. Subsequently, α-W is transformed into γ-W. Notably, phase transformation only occurs under certain irradiation conditions. Two types of phase transformation are illustrated in Figs 12 and 13. The detailed process of these two phase transformations will be described in the following sections.

The bcc structure of α-W is highly stable; therefore, the phase transformation requires high-intensity ion irradiation. Under high He-ion irradiation conditions, the induced defects of interstitial atoms and the formed He bubbles will release the internal stress, thereby resulting in lattice distortion. From our viewpoint, when the interstitial atoms or the He bubbles satisfy certain conditions, the process of phase transformation will occur.

**Figure 14:**
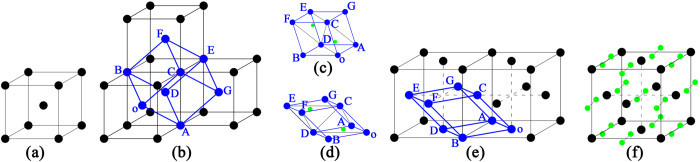
Model of the first process of phase transformation.

The phase transformation from α-W to γ-W may be due to the lattice distortion, which is driven by thermal diffusion of He atoms at high temperature (1377 K) when the He atom occupies the TIS in the W lattice. The detailed processes are elaborated by the atomic lattice model. As shown in Fig. 14a–f, a concise model was constructed to illustrate the process of phase transformation. A bcc structure of the α-W unit cell, which is presented in Fig. 14a, was selected from JCPDS #04-0806, which has a lattice constant of approximately 3.1648 Å. Four unit cells are stacked, as shown in Fig. 14b, and form a monoclinic lattice, which is indicated by the blue balls and lines. The bcc structure of α-W could be considered a periodically arranged monoclinic cell, where lattice constant parameters *a*, *b*, and *c* correspond to the angles between edges of *α*, *β*, and *γ* of the monoclinic lattice of approximately *a* = *b* = *c* = 2.7408 Å, *α* = *β* = 70.53°, and *γ* = 109.47°. Meanwhile, the fcc structure of γ-W is indexed on the basis of JCPDS #88-2339, with a lattice constant of approximately 4.06 Å. A monoclinic lattice could also be observed in a two-unit cell, as shown in Fig. 14e. After calculation, the parameters of the lattice constants *a*, *b*, and *c* and the angles between edges of *α*, *β*, and *γ* of the monoclinic lattice of approximately *a* = *b* *=* *c* = 2.8709 Å are *α* = *β* = 60°, *γ* = 90°. In the phase-transformation process, the lattice constant only requires a change of 0.1301 Å, whereas the angles between edges of *α* = *β* and *γ* must be changed to 10.53° and 19.47°, respectively. Thus, the phase transformation changes the angles and lattice constants. When two He atoms are inserted into the monoclinic lattice and occupy the TIS (as shown in Fig. 14c), these structures cover a larger region. In this structure, each He atom will react with the neighboring He atoms^48^,^49^, resulting in lattice distortion due to TIS relaxation. The binding energy is approximately 1 eV^49^ between two He atoms. Overcoming this binding energy results in the formation of a He–He cluster. At high temperature (1377 K), the tetrahedral interstitial He atoms have sufficient migration energy to form a stable structure of He–He clusters via thermal diffusion. In the monoclinic lattice, the nearest-neighbor He atom is at 0.5*a*_0_ = 1.5824 Å. The nearest neighbor He atoms have a tendency to migrate and form a He–He cluster^49^. At a temperature of 1377 K, the migration path of He diffusing in W should be a mixture of the TIS–TIS and TIS–OIS–TIS paths^51^,^52^; i.e., the nearest-neighbor He atoms will be close to the OIS. For the triangular pyramid o–ADC, the He atom is close to the central of the rhombus of oADB, resulting in the elongated edge of oD with a shortened AB edge. For the rhombus of oCGA, the edge of AC shortens. The other He atoms have equivalent results for the other triangular pyramid, E–FCD. The process would lead to a decrease of the angles *α*, *β*, and *γ* and to an adjustment of the edges. After full relaxation, stable configurations would be obtained, as displayed in Fig. 14d. This change may yield the stable structure shown in Fig. 14e. A new fcc structure, similar to that in Fig. 14f, would be obtained after this lattice distortion. The result of the lattice distortion is the phase transformation from α-W to γ-W. In the fcc unit cell of γ-W, each OIS is set with two interstitial He atoms to obtain a stable structure of a He–He cluster, resulting in an increase of the interplanar spacing. In addition, each fcc unit cell consists of four W atoms and contains eight interstitial He atoms, which corresponds to a monoclinic lattice consisting of one W atom and containing two interstitial He atoms. On the basis of the model, a relationship exists between α-W and γ-W that follows the crystallographic relation {011}_α_//{001}_γ_; {110}_α_//{–111}_γ_; {101}_α_//{111}_γ_. This model can well explain the phase transformation, which is reduced by the interstitial He atoms occupying the TIS and causing the lattice distortion. In Fig. 12, the HRTEM image (Fig. 12a) coincides to the model at the zone axis of [10–1] and to the crystallographic relation of (101)_α_//(111)_γ_. The interplanar spacing of γ-W is increased, which is in good agreement with the He atoms set in the OIS (Fig. 14f). In this phase transformation, the He atoms must occupy the particular TIS. Such conditions are difficult to satisfy on a large scale under He-ion irradiation. Thus, this phase transformation only occurred at some small areas.

**Figure 15:**
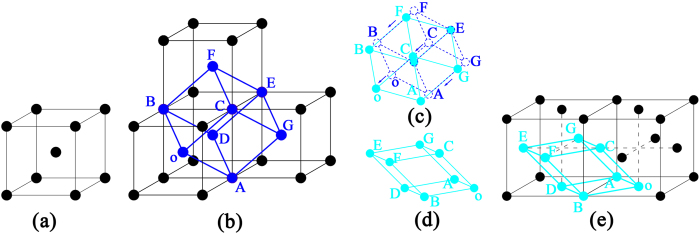
Model of the second process of phase transformation.

Under high fluence, He-ion irradiation increases the temperature to 1377 K, thereby enabling the He clusters or bubbles to form and grow into the large bubbles by trapping He atoms. The He bubbles with high internal stress (shear strain energy)^46^ affect the surrounding W atoms, thereby resulting in the other method of phase transformation from α-W to γ-W by shear strain. This process is illustrated by the atomic lattice model in Fig. 15a–e. Figure 15a,b are the same as Fig. 14a,b, which show a monoclinic lattice. Figure 15c describes the shear process, showing that the D and E atoms remain intact. The A, B, F, and G atoms migrate with a unit displacement of 0.5218 Å in the plane (211) along the [1–1–1] direction, and the o and C atoms migrate with two unit displacements of 1.0436 Å in the same plane and in the same direction of [1–1–1]; i.e., the shear process occurs at the (211) plane and along the [1–1–1] direction. After the lattice slightly adjusts in size and angle, a stable new monoclinic lattice is obtained (similar to that in Fig. 15d), which agrees well with the monoclinic lattice denoted by cyan balls and lines in Fig. 15e. Subsequently, the phase transformation from α-W to γ-W is completed. The phase transformation from α-W to γ-W occurs when the shear strain energy is sufficiently large to overcome the relevant energy barrier. In this model of phase transformation, the shear direction of [1–1–1] is the close-packed direction of 〈111〉 for the bcc structure W, which implies a low slip force could induce the (211) plane slip along this direction. The He bubbles can induce the most stable 〈111〉-dumbbell self-interstitial atom (SIA)^46^,^53^. Kong *et al.*^54^ proposed that the 〈111〉-dumbbell SIA spontaneously forms the stable structure of 〈111〉-crowdion. Thus, the process of migration of W atoms along the [1–1–1] direction may involve motion of W atoms from their original positions to the 〈111〉 interstitial sites, thereby forming a stable 〈111〉-crowdion structure. Henriksson *et al.*^55^ reported that the He bubble growth could yield the 〈111〉-crowdion interstitials that migrate to the surface. This migration leads to displacement of W atoms by approximately 2.7 Å in the [111] direction. Thus, this method of phase transformation is likely to occur when a large bubble acts on the surrounding W atoms. In this model of phase transformation, the (211) plane of the α-W phase is the no-distortion plane, which exhibits no obvious distortion and no rotation during the process of phase transformation. In addition, the corresponding plane for the γ-W phase is the (011) plane. The (211) plane of atoms begins to undergo shear motion along the [1–1–1] direction under the shear stress induced by the He bubbles; the corresponding direction to the γ-W is the [01–1] direction. Thus, this model reveals a relationship between α-W and γ-W that follows the habitus relation of {211}_α_//{011}_γ_; 〈111〉_α_//〈011〉_γ_. This model should be used to illustrate the second type of phase transformation shown in Fig. 13. In the model, the He bubble is located at the plane of (011) for the α-W phase along the [100] direction. The position of the He bubble remains unchanged during the course of the phase transformation. Thus, when the incident electron is along the [110] direction for the γ-W phase, the position of the He bubble is the (–111) plane. These results agree with the relationship shown in Fig. 13c. For this mode of phase transformation induced by shear stress, the results are the formation of He clusters or He bubbles and the lack of detection of an obvious distortion in the structure. These results are consistent with the HRTEM images in Fig. 13b,c. The phase transformation model can be used to explain the second process of phase transformation resulting from the He bubbles inducing W-atom migration along the 〈111〉 direction.

### Phase transformation effect on fusion W materials in the ITER

The phase transformation of W is speculated to significantly affect the fusion processes of W materials. In our study, the phase transformation of W is caused by high-fluence He irradiation and the transformation can proceed via one of the two mechanisms. The corresponding stress fields partially relax, leading to the phase transformation. First, the He ions enter the TIS and migrate and diffuse at high temperature, thus reducing the lattice distortion and leading to the phase transformation. Second, the He cluster or bubble can yield shear stress and induce the (211) plane to migrate along the 〈111〉 direction, which relieves the stress via phase transformation. No evident boundaries are observed between α-W and γ-W, and insufficient internal stress is observed in the boundary. However, performing studies to understand which structure of the bcc-structured α-W and fcc-structured γ-W exhibits better anti-radiation performance, as well as the corresponding behavior of irradiation, is critical. In general, because the fcc structure is a close-packed structure, it should exhibit greater irradiation resistance than the bcc structure. In a complex and severe fusion environment, every change is important, making the study of the phase transformations in W highly significant.

## Methods

**Figure 16:**
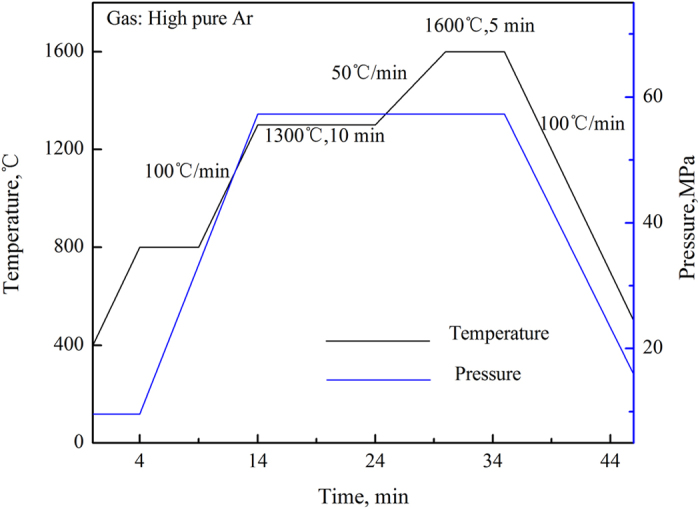
Temperature and pressure profiles of the SPS process of the W–Y_2_O_3_ alloy.

W–Y_2_O_3_ composite powder with 2 vol.% W–Y_2_O_3_ content was fabricated using ammonium para-tungsten (APT, purity: ≥99.95%) and yttrium nitrate hexahydrate (Y(NO_3_)_3_∙6H_2_O, purity: ≥99.5%) via a new chemical method. The two chemicals were dissolved in deionized water to form a transparent solution. Oxalic acid (C_2_H_2_O_4_∙2H_2_O) was used to precipitate the solution, which was placed in a methyl silicone oil bath at 185 °C until the precursor was obtained. After being ground, the precursor was placed in a tubular furnace and reduced by high-purity hydrogen. The reduced powder was consolidated by spark plasma sintering (SPS) at a sintering temperature of 1600 °C for 5 min; the sintering curve is shown in Fig. 16. The diameter and thickness of the sample were approximately 20 and 2.5 mm, respectively.

The density of the samples was measured using Archimedes’ immersion method, and the relative densities were calculated from the volume fraction; theoretical densities of tungsten and W–Y_2_O_3_ were adopted as 19.25 and 5.01 g/cm^3^, respectively. The Vickers microhardness of the specimens before and after irradiation was measured on polished sections and on the irradiated surface, respectively, using an MH-3L microhardness tester along the surface of the specimen, with a load of 0.49 N (50 gf) maintained for 15 s; 10 readings were collected and averaged for each tested section.

**Figure 17:**
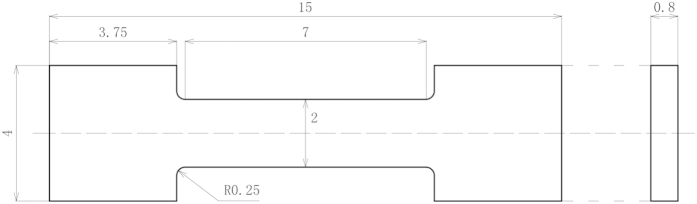
Dimensions of the tensile specimens.

Before the specimens were irradiated, tensile and nano-indentation tests were performed. Small, smooth specimens were tested at room temperature (RT), 400, 600, and 800 °C using an INSTRON-5967 testing machine. The dimensions of the small specimens are shown in Fig. 17. Nano-indentation tests were performed using an Agilent nano-indenter G-200 and a Berkovich tip, which is a three-sided pyramid. Indentation was conducted under various loads to study the hardness as a function of load.

Mirror-quality polished W–Y_2_O_3_ plates with dimensions of 10 × 10 mm^2^ were maintained at RT and irradiated with He-ion beams for 2 h using the Large-Power Materials Irradiation Experiment System (LP-MIES). The corresponding beam energy was 80 eV, and the ion dose was approximately 8.64 × 10^25^ ions/m^2^ during the implantation experiments. The He-ion beams were incident perpendicular to the sample surface and with a 10-mm effective circular region. During He-ion irradiation, the temperature of the targets was measured using an infrared STL-150B pyrometer. The He-ion flux remained constant at 1.2 × 10^22^ ions/(m^2^s), resulting in a surface temperature of 1377 K ± 10 K.

XRD patterns were acquired to verify the purity of the obtained powders and to determine the phase stability of the samples after irradiation. The morphologies of the surface and the fracture of the W–Y_2_O_3_ sample were examined using field-emission scanning electron microscopy (FE-SEM, SU8020, Japan); the microscope was equipped with an energy-dispersive X-ray spectrometer. The interface between the irradiated and non-irradiated sites was also characterized. The microstructure of the W–Y_2_O_3_ alloy was observed using transmission electron microscopy (TEM, JEM-2100 F, Japan). A disc with diameter of 3 mm and thickness of approximately 500 μm was cut from the W–Y_2_O_3_ alloy sample using a linear cutting machine. The disc was then ground using SiC papers with grades ranging from 120 to 1200. After the disc was polished, its thickness was approximately 50 μm. The disc for TEM was prepared using ion-thinning technology (model 691, Gatan).

The sample was characterized after irradiation. For structural characterization, the surface of the irradiation-damaged region was scraped with a knife, placed in ethanol solution, and then dispersed using ultrasonic waves. The sample was then analyzed using TEM. HRTEM images were obtained to clarify the process of irradiation damage. Crystallographic analysis was used to analyze the changes of the lattice structure. For mechanical characterization, the sample was subjected to Vickers microhardness tests to analyze the irradiated surface, the non-irradiated surface, and the interface between the irradiated and non-irradiated regions.

## Conclusion

In this work, W–Y_2_O_3_ alloys were prepared through a wet-chemical route and powder metallurgy. W–Y_2_O_3_ alloys have not only excellent mechanical performance but also excellent plasticity. After irradiation, the material surface exhibited nanosized fuzz and a thin layer of damaged subsurface. Notably, irradiation embrittlement was reflected in the evident cracks in the indentations at the irradiation region. Lattice distortion, polycrystallization and amorphization were observed to be the mechanisms by which the internal stress induced by irradiation was relieved. The phase transformation from the bcc structure of α-W to the fcc structure of γ-W was also a method to relieve the internal stress, as was confirmed by XRD and TEM analyses. The mechanisms of phase transformation were clearly determined in this study: one mechanism is caused by the He atoms occupying the TIS and diffusing to the OTS to form He–He clusters; the other mechanism involves He bubble or cluster yield shear strain.

## Additional Information

**How to cite this article**: Tan, X. *et al.* Mechanical properties and microstructural change of W-Y_2_O_3_ alloy under helium irradiation. *Sci. Rep.* **5**, 12755; doi: 10.1038/srep12755 (2015).

## Figures and Tables

**Table 1:** Tensile test results for the W–Y_2_O_3_ alloy at different temperatures.

**Temperature**	**Yield strength δ**_**0.2**_ **(MPa)**	**Tensile strength (MPa)**	**Elongation (%)**	**Elastic modulus**
400	397.4	436	6.4	6.65
600	273.1	346	7.1	5.0
800	206.4	249	3.9	4.5

**Table 2:** Microhardness of the W–Y_2_O_3_ alloy.

**Areas**	**Non-irradiated area**	**Irradiated region**	**RS-irradiated area**	**Affected area**
Hv, MPa	338.4	288.5	330.4	328.1–365.8
